# Antioxidant, Antihypertensive and Antimicrobial Properties of Phenolic Compounds Obtained from Native Plants by Different Extraction Methods

**DOI:** 10.3390/ijerph18052475

**Published:** 2021-03-03

**Authors:** Francisco Ramiro Boy, Rocío Casquete, Ana Martínez, María de Guía Córdoba, Santiago Ruíz-Moyano, María José Benito

**Affiliations:** Nutrición y Bromatología, Instituto Universitario de Investigación en Recursos Agrarios (INURA), Escuela de Ingenierías Agrarias, Universidad de Extremadura, 06007 Badajoz, Spain; ramboy1@hotmail.com (F.R.B.); amartinehi@alumnos.unex.es (A.M.); mdeguia@unex.es (M.d.G.C.); srmsh@unex.es (S.R.-M.); mjbenito@unex.es (M.J.B.)

**Keywords:** phenolic compounds, dehesa plants, functional properties, antimicrobial activity

## Abstract

This study aims to evaluate the efficacy of two methods (agitation and ultra-sound) for extracting phenolic compounds from 15 native plants. Plant species collected in the Dehesa of Extremadura were used. The antioxidant, antihypertensive and antimicrobial activity of the phenolic extracts was investigated. Significantly different results were obtained when comparing the two extraction methods, with the highest concentrations of phenolic compounds found for ultrasound extraction. In addition, the extracts obtained for *Cistus albidus*, *Cistus salviifolius*, *Rubus ulmifolius* and *Quercus ilex* showed the highest concentrations of phenolic compounds. The antioxidant activity was higher in the extracts of *Cistus* and *Q. ilex* obtained by ultrasound, as was the antihypertensive activity. Antimicrobial activity was also higher in the extracts obtained by ultrasound from *C. salviifolius* and *Q. ilex* plants against bacteria and from *Cistus ladanifer* against yeasts. Therefore, it can be concluded that, with the ultrasound extraction of phenolic compounds from *C. ladanifer*, *C. albidus* and *Q. ilex* plants, it is possible to obtain extracts with important functional properties, so they could be studied for their use in food with the aim of obtaining healthy and safe products, favouring the sustainability of the environment of the Dehesa Extremeña.

## 1. Introduction

Nowadays, the importance of aromatic and medicinal plants is recognized worldwide, not only for their curative and preventive properties, but also for their uses in the pharmaceutical and food industries [1]. The Dehesa of Extremadura is characterized as an ecosystem with great biodiversity where many species of plants considered to be medicinal can be found, and are important in the pharmaceutical industry [2,3]. Among these medicinal plants are *Lavandula stoechas*, *Malva sylvestris*, *Rosmarinus officinalis* [4], *Cistus ladanifer*, *C. multiflorus*, *C. albidus* and *C. salviifolius* [5]. In addition to their medicinal properties, some of these plants, such as *L. stoechas* and *M. sylvestris*, are aromatic, which makes them excellent condiments for meals. However, their aroma is not the only benefit in food; their antioxidant and antimicrobial nature is also important. They can compete with chemical antioxidants that have been associated with possible health consequences (allergic reactions, disorders in pregnant women and children, possible carcinogenic action, etc.) [6,7].

The functional properties of these plants, such as their antioxidant or antimicrobial properties, are due to the phenolic compounds present in their fruits, seeds, leaves, stems and flowers [8]. Phenolic compounds constitute a wide group of compounds that result from the secondary metabolism of plants found in different natural sources. The molecular structure of phenolic compounds has an aromatic ring containing one or more hydroxyl groups and can be a single or polymerized molecule. Depending on their structural characteristics, phenolic compounds are divided into several groups, although the main ones are phenolic acids, flavonoids, and non-flavonoids [9].

It is considered that the antioxidant properties of these compounds are due to their redox properties and chemical structure, responsible for neutralizing free radicals, chelating metals, and decomposing peroxides [10,11,12]. This beneficial property retards the development of diseases related to oxidative stress such as cancer, diabetes, and Alzheimer’s disease [13,14], as well as anti-inflammatory activity [15]. In addition, a protective role against oxidation of unsaturated lipids in food has been observed, mainly in meat and meat products [16,17].

In addition, recent studies have documented the antibacterial effects of phenol-rich extracts commonly found in plants. These studies have highlighted the use of phenolic compounds to control pathogenic bacteria, including those with a commercial antibiotic resistance profile [18,19]. The antibacterial effects of phenolic compounds have also been primarily associated with the presence of hydroxyl groups in their molecules. In fact, the number and position of these hydroxyl groups, i.e., the pattern of hydroxylation, in the phenolic ring, seems to be associated with the importance of the inhibitory effects exerted by the phenolic compounds on the target bacteria [20,21]. A bactericidal effect has been proven against foodborne bacteria such as *Escherichia coli*, *Salmonella* sp., *Listeria monocytogenes* and *Staphylococcus aureus* [18,22,23].

Extracts obtained from plants have also been reported for their antihypertensive activity [24], although there are not many recent studies on this activity in aromatic plants. Hypertension is one of the most important factors contributing to cardiovascular diseases; therefore, it is very important to find natural compounds with this activity. Vargas-León et al. [25] attributed the antihypertensive activity of *Hibiscus sabdariffa* to the high number of phenolic compounds it possesses, with flavonoids and anthocyanins being the main components.

To study the biological activity of extracts, the extraction methods used are crucial since they determine the purity grade at which the phenolic compounds are extracted, and at the same time, affect all their beneficial properties previously mentioned. Thus, there are several extraction methods, including agitation and ultrasound, which are simple and cost-effective methods. In both methods, it is necessary to use a suitable solvent to extract these compounds, among which are ethanol (70–80%), water and methanol, the last-mentioned being used in different concentrations as well as ethanol [26].

This research is an exploratory work for plant selection with the best characteristics. Therefore, this study aims to evaluate which of the two methods (agitation or ultrasound) is more effective in the extraction of phenolic compounds of different scarcely studied native plants obtained from the Extremadura Dehesa, and their influence on the functional antioxidant, antihypertensive and antimicrobial properties of the extracts obtained. The selected functional extracts could be studied for their use in food with the aim of obtaining healthy and safe products, favouring the sustainability of the Dehesa Extremeña environment.

## 2. Materials and Methods

### 2.1. Plant Material

For the development of the work, the leaves, stems, and flowers of 15 plant species (flowers from *Calendula officinalis*, stems from *Cistus ladanifer*, *Cistus multiflorus*, *Cistus albidus*, *Cistus salviifolius*, *Lavandula stoechas*, *Crataegus monogyna*, *Malva sylvestris* and *Asparagus* sp., and leaves from *Rosmarinus officinalis*, *Rubus ulmifolius*, *Quercus ilex*, *Morus alba*, *Ulmus* sp. and *Urginea maritima*) were used. The plants were harvested from the Extremadura region of Spain by a company located there. The samples were vacuum-packed in plastic bags and stored at −20 °C until they were used to extract the bioactive compounds.

### 2.2. Bacterial Strains

Foodborne pathogenic bacteria and spoilage yeasts in food obtained from the Spanish type culture collection (CECT) were used to run the study. The pathogenic microorganisms were *Staphylococcus aureus* CECT 976, *Bacillus cereus* CECT 131, *Listeria monocytogenes* CECT 911, *Listeria innocua* CECT 910, *Salmonella choleraesuis* CECT 4395, *Escherichia coli* CECT 4267, *Candida boidinii* CECT 11,153, *Priceomyces carsonii* CECT 10,230, *Kregervanrija fluxuum* CECT 12,787 and *Zygosacharomyces bailii* CECT 11,043.

### 2.3. Phenolic Compound Extraction

The phenolic compounds were extracted from the plants by mechanical agitation (AE) and ultrasound-assisted extraction (UE).

#### 2.3.1. Mechanical Agitation Extraction

Each sample (10 g) was extracted with 60 mL of 90% (*v*/*v*) ethanol–water extraction solution using an orbital shaker (Thermoshake THO 500, Gerhardt Analytical Systems, Konigswinter, Germany) set at 120 rpm and 25 °C for 2 h in darkness. After the 2 h of extraction, the samples were filtered. This process was repeated twice. The extracts were then kept in the dark for 24 h at 4 °C. After that, excess ethanol was removed by heating at 37 °C in a rotary evaporator under vacuum (Heildolph mod. Hei-VAP Precisión, Heidolph, Germany). The resultant aqueous extracts were combined to a final known volume and stored at −20 °C until analysis.

#### 2.3.2. Ultrasound-Assisted Extraction

Each sample (10 g) was mixed with 60 mL of ethanol (90% *v*/*v*) and placed inside an ultrasonic bath (360 W, J.P. Selecta, s. a. Barcelona, Spain). The samples were sonicated by ultrasound at a frequency of 50/60 Hz and power of 220 V for 2 h in the absence of light at room temperature. After the 2 h of extraction, the samples were filtered. This process was repeated twice. The extracts were then kept in the dark for 24 h at 4 °C. After that, excess ethanol was removed by heating at 37 °C in a rotary evaporator under vacuum (Heildolph mod. Hei-VAP Precisión). The resultant aqueous extracts were combined to a final known volume and stored at −20 °C until analysis.

### 2.4. Total Phenolic Content (TPC)

The TPC of the above stored extracts was determined using Folin–Ciocalteu reagent and the method described by Wettasinghe and Shahidi [27] in a UV-1800 spectrophotometer (Shimadzu Scientific Instruments, Columbia, MD, USA). Gallic acid was used as standard. Results are expressed as mg of gallic acid equivalents (GAE)/100 g of fresh plants. All experiments were conducted in triplicate.

### 2.5. Antioxidant Activity by Free Radical Scavenging Ability Using a Stable DPPH Radical and ABTS Radical Cation

The antioxidant activity of the above stored extracts was determined by bleaching of the purple-coloured solution of 1,1-diphenyl-2-picrylhydrazyl radical (DPPH) according to the method of Teixeira et al. [28]. The total antioxidant activity was expressed as mg of Trolox/100 g of fresh plants. All experiments were conducted in triplicate.

The free radical scavenging capacity of extracts was also determined using the ABTS (2,2′-azino-bis-(3-ethylbenzothiazoline-6-sulfonic acid)) radical cation decolorization assay, according to the procedure proposed by Cano et al. [29], slightly modified (*n* = 3). The initial absorbance value at λ 730 nm was then compared to the absorbance obtained after 20 min of reaction. The results were expressed as mg of Trolox/100 g of fresh plants.

### 2.6. Assessment of ACE Inhibitory Activity

The extracts were diluted in 40% methanol (*v*/*v* water) at a concentration of 400–100 µg/mL, then 1:2, 1:4 and 1:8 dilutions with Milli-Q water were done. Angiotensin-converting enzyme (ACE) inhibition activity was determined according to the method developed by Sentandreu and Toldrá [30,31] with some modifications. ACE, previously dissolved in 50% glycerol, was diluted in 0.15 M Tris buffer (pH 8.3) containing 0.1 mM ZnCl_2_ with 0.04 U/mL of enzyme in the final reaction solution. Into 96-well microplates (black polystyrene, Porvair, Leatherhead, UK), 40 μL of distilled water or the ACE working solution was added. Then, the reaction mixture was adjusted to 80 μL by adding distilled water to the blank (B), control (C) or sample (S). A sample blank (SB) was prepared by substituting distilled water for the sample to take into consideration the interference of the compounds. The reaction was begun by adding 160 μL of 0.45 mM o-Abz-Gly-p-Phe (NO_2_)-Pro-OH (Bachem Feinchemikalien, Bubendorf, Switzerland) dissolved in 150 mM Tris-base buffer (pH 8.3), with 1.125 M NaCl, and was incubated at 37 °C. The fluorescence was registered at 30 min using a multiscan microplate fluorimeter (FLUOstar optima, BMG Labtech, Offenburg, Germany). The emission and excitation wavelengths were 420 and 350 nm, respectively, and data were processed using FLUOstar control (version 1.32 R2, BMG Labtech). The activity of each sample was tested in triplicate. Inhibitory activity is defined as the compound concentration required to inhibit the original ACE activity. The formula applied to determine the percentage of ACE inhibitory activity was: 100 × [(C − B) − (S − SB)]/(C − B).

Then, the concentration of 50% inhibition (IC50) was obtained by linear interpolation from the graphical representation of the fractional activity vs. the phenolic extract concentration. IC50 is the phenolic concentration (µg/mL) required to decrease ACE activity by 50% under the assayed conditions, graphically obtained where Vi inhibitor/Vi control = 0.5.

### 2.7. Antimicrobial Activity

Target cell suspensions (Section 2.2) were prepared from cultures incubated overnight at 37 and 25 °C on brain–heart infusion agar (BHI; Oxoid, Madrid, Spain) and yeast peptone and dextrose extract agar (YPD; Oxoid, Madrid, Spain) for the bacteria and yeast, respectively. After the incubation time, colonies were transferred to a sterile Peptone Water solution (Scharlab, Barcelona, Spain) to obtain a turbidity equivalent to 0.5 McFarland standards. Next, 1 mL of each suspension was pipetted into separate sterile petri dishes to which 20 mL of molten BHI and YPD with 1% agar (45 °C) for the bacteria and yeast, respectively, were added. Once set, 10 μL of aqueous extracts at different concentrations (2, 1 and 0.5 mg/mL) was added. Sterile distilled water, instead of active compounds, was used as the negative control. The plates were incubated overnight at 37 and 25 °C for the bacteria and yeast, respectively, and the diameter (mm) of the resulting inhibition zone was measured. All experiments were conducted in triplicate.

### 2.8. Statistical Analysis

Statistical analysis of the data was carried out using SPSS for Windows, version 21.0 (SPSS Inc., Chicago, IL, USA). Descriptive statistics of the data were determined, and the differences within and between groups were studied by one-way and three-way analysis of variance (ANOVA) and separated by Tukey’s honest significant differences test (*p* < 0.05). In addition, principal component analysis (PCA) on the correlation matrix of the variables was performed using SPSS for Windows, 203 version 21.0 (SPSS Inc., Chicago, IL, USA).

## 3. Results and Discussion

The total phenolic compounds obtained by the two extraction methods used, agitation and ultrasound, from the different plants used in the study are presented in Table 1.

In general, when the two extraction methods were compared, it was found that there were significant differences, the highest values being found for the ultrasound extraction. The concentrations of phenolic compounds obtained by ultrasound ranged from 57.94 to 1260.47 mg GAE/100 g, and those obtained by agitation ranged from 26.95 to 1029.25 mg GAE/100 g. This agrees with the results reported by Bimakr et al. [32] in which ultrasound extraction allowed for the acquisition of 152.25 mg GAE/g from *Malva sylvestris*, while only 128.88 mg GAE/g was obtained by agitation. In a different research, the ultrasound extraction efficiency was also higher, showing a maximum value of 16.41 mg GAE/g of blueberry pomace, while the conventional agitation method showed a maximum value of 5.08 mg GAE/g [33]. Due to the above, it can be said that extraction by ultrasound proved to be more effective.

Furthermore, comparing the plants it can be observed that *Cistus albidus*, *C. salviifolius*, *Rubus ulmifolius* and *Q. ilex* were the ones with the highest phenolic compound concentrations obtained, the levels being between 927.84 mg and 1260.47 mg GAE/100 g (*p* < 0.05). *Cistus* is an aromatic plant highly valued for its functional properties, among which the phenolic compound content is remarkable. In the study performed by Abu-Orabi et al. [34], the concentrations of phenolic compounds in the flowers of *C. salviifolius* ranged from 111 to 183.8 mg GAE/g, and in the leaves from 126 to 393 mg GAE/g. In our study, one of the plants that presented more phenolic compounds was *Q. ilex,* corroborating with many studies that have demonstrated a high content of these bioactive compounds, as presented by Amessis-Ouchemoukh et al. [35]. Hadidi et al. [36] also obtained between 2103.346 and 4580.316 mg GAE/100 g from the leaves of this tree.

The antioxidant activity analysed by the two methods, DPPH and ABTS, and the antihypertensive activity determined by the ACE inhibitory activity of the extracts obtained from the plants are presented in Table 2.

In general, it can be observed that the ABTS values were higher than those provided by the DPPH method (Table 2), which may be related to the way in which the ABTS and DPPH radicals work. However, comparing the compound extraction methods, it was found that the values obtained by ultrasound were higher than those found for agitation (*p* < 0.05): between 91.23 and 2363.34 mg Trolox/100 g for DPPH and 154.62 and 2306.17 mg Trolox/100 g for ABTS. In addition, when analysing the activity according to the plants, *Cistus* and *Q. ilex* were the ones with the highest values, meaning that those plants showed the highest antioxidant activity. Furthermore, it can be observed that the extracts with the highest antioxidant activity were not those with the highest total phenolic compound concentrations, which may be due to the different compound compositions [37,38]. The genus *Cistus* is one of the plants with greater beneficial properties, among which its antioxidant activity, related to its phenolic compound content extracted mainly with ethanol is highlighted [39]. Among the species highlighted for their antioxidant character is *Q. ilex,* which is reported in this study and supported by other studies such as that by Arina and Harisun [40], who obtained 90% radical inhibition.

Hypotensive activity, expressed as IC50, measures the amount of any substance needed to inhibit 50% of ACE. Table 2 shows that the phenolic compounds extracted from *Cistus multiflorus*, *Malva sylvestris*, *R. ulmifolius*, *Q. ilex*, *Morus alba* and *Urginea marina* using ultrasound had a lower IC50 (*p* < 0.05) than those extracted by agitation. This means that, with a smaller amount of these compounds, the ACE activity is reduced to half and ultrasound is more effective as an extraction method. However, within the plants, it can be observed that species of the genus *Cistus*, among them *C. albidus*, *C. ladanifer* and *C. salviifolius*, were the ones with a lower IC50, with values between 5.85 µg/mL (*C. ladanifer*) and 21.69 µg/mL (*C. albidus*). Additionally, *R. ulmifolius*, with an IC50 of 65.40 µg/mL, was also found within these plants with lower IC values. This activity is directly related to the phenolic compound content, so that the higher the amount of these bioactive compounds, the higher the ACE inhibitory activity. This fact has been confirmed in this study and in others such as the one presented by Chaudhary et al. [41] on several medicinal plants.

Table 3 shows statistical analysis of the variables studied (plants, extraction method and concentration) and the antimicrobial effect on the six pathogenic bacteria analysed, as well as their interactions (*p* < 0.05).

Concerning the study of the plants’ effect, it can be observed that *C. salviifolius* and *Q. ilex* presented a greater inhibition capacity on the six pathogenic bacteria studied (*p* < 0.05). The rest of the plants belonging to the genus *Cistus* (*C. multiflorus*, *C. albidus* and *C. ladanifer*) presented a greater inhibition capacity against *Salmonella choleraesuis*. *R. ulmifolius* presented a greater inhibition capacity against *Listeria monocytogenes*, *S. aureus* and *E. coli*.

Regarding the extraction method, greater inhibition capacity was obtained through ultrasound against three of the six bacteria studied: *L. monocytogenes*, *L. innocua* and *S. choleraesuis*.

The concentration of extract also affected this activity, since the higher its concentration, the higher its inhibition capacity, with 2 mg/mL being the concentration with the greatest antimicrobial capacity against the six bacteria studied. Therefore, the genus *Cistus* is highlighted for its antimicrobial capacity. This agrees with the results obtained by Mahmoudi et al. [42] who, after studying the antimicrobial activity of two species of the genus *Cistus*, observed higher activity against *E. coli*. It is commonly known that Gram-positive bacteria are more susceptible to natural extracts and Gram-negative ones are less sensitive to natural extracts. Nevertheless, there are exceptions in which Gram-negative bacteria are more susceptible than Gram-positive ones towards some natural extracts [43], highlighting the susceptibility of *E. coli*.

In addition, *Quercus* extract contains metabolites belonging to various chemical groups; the phenolic compounds (benzoics, cinnamics, coumarins, stilbenes, flavonoids, lignans and tannins) are the most representative. Other groups include carbohydrates, amino acids, fatty acids, carboxylic acids, and other secondary metabolites, terpenoids and alkaloids [44]. Some of these compounds have been previously reported as antimicrobial compounds in *Q. ilex* and another *Quercus* spp. [45]. The antimicrobial activity of this genus has been proven against various microorganisms, including *E. coli* and *S. aureus*. This activity depends on the species and the extract obtained, as well as the method of acquisition [46].

Based on the results, it is possible to state that the antibacterial activity is influenced by the method of extracting phenolic compounds, the concentration and the plant extract used.

Table 4 shows the statistical analysis of the variables studied (plants, extraction method and concentration) and the antimicrobial effect on the four spoilage yeasts analysed, as well as their interactions (*p* < 0.05).

*Cistus ladanifer* showed a greater inhibition capacity against the four studied spoilage yeasts and *C. albidus* showed greater capacity against *K. fluxuum*, *P. carsonii* and *Z. bailii* (*p* < 0.05; Table 3). A greater inhibition capacity of *Q. ilex* can also be observed against *C. boidinii*, *K. fluxuum* and *Z. bailii*.

In addition, greater inhibition capacity against the studied yeasts was obtained with ultrasound extraction, and the highest concentration of extracts, 2 mg/mL, showed a greater antimicrobial capacity.

Ceylan et al. [47] performed studies with several aromatic plants and observed their activity against yeasts of the genus *Candida*.

When comparing the results presented in Table 3 and Table 4, it can be observed that plants with activity against bacteria do not show activity against yeasts or do so to a lesser extent. That was the case for *C. salviifolius*, *R. ulmifolius* and *Malva sylvestris*. The first two presented lower activity against yeasts compared to bacteria (Table 3); *M. sylvestris* did not present any activity against the studied yeasts, so it was not included in Table 4.

*Q. ilex* is also described as having antimicrobial and antifungal properties. Merghache et al. [48] demonstrated the inhibition capacity of the wood ashes of *Q. ilex* against yeasts even at low concentrations (5%).

Therefore, as previously stated for antibacterial activity, based on the results, antifungal activity is influenced by the method of extracting phenolic compounds, the concentration and the plant extract used.

**Figure 1:**
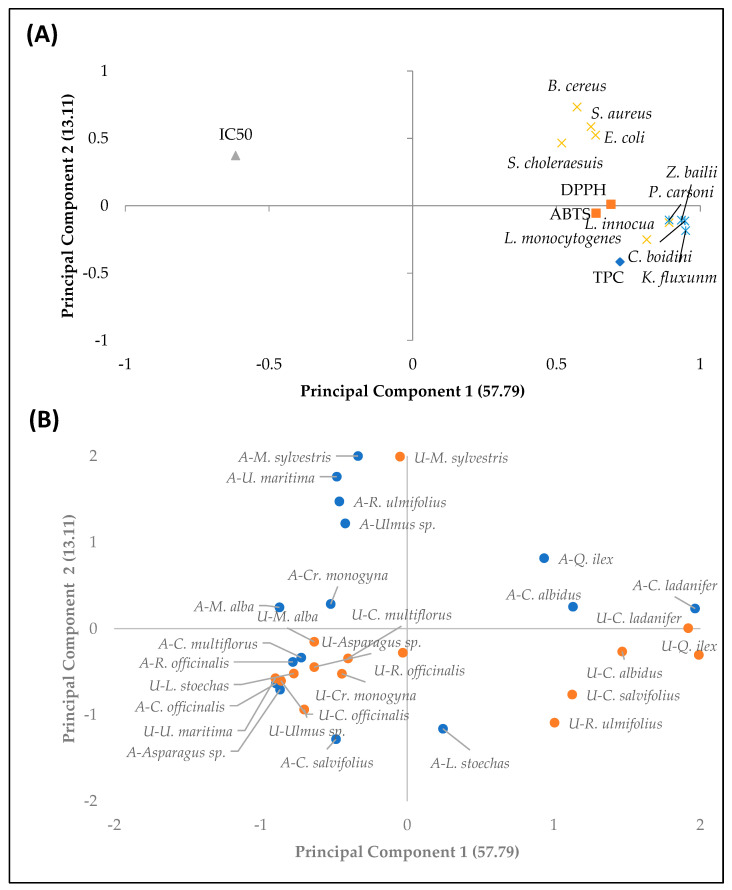
Principal component analysis of the parameters analysed in the extracts from different plants extracted by agitation (A) and ultrasound (U). Loading plot (**A**). TPC: total phenolic compounds; DPPH and ABTS: antioxidant activity of extracts; IC50: extract concentration (µg/mL) required to reduce 50% of ACE; *B. cereus*, *S. aureus*, *E. coli*, *S. choleraesuis*, *L. innocua* and *L. monocytogenes*: antimicrobial activity against each of the bacteria; *Z. bailii*, *P. carsonii*, *C. boidinii* and *K. fluxuum*: antimicrobial activity against each of the yeasts. Score plot (**B**). Plant extracts extracted by agitation (blue); plant extracts extracted by ultrasound (orange).

Finally, the PCA developed with the different parameters studied, to determine the relevance of each parameter in the plant extracts obtained with the two extraction methods used, is shown in Figure 1. 

Figure 1 shows that the *Q. ilex* extracts extracted by ultrasound (U) and those of *C. ladanifer* extracted by both methods (A and U) are located on the negative and positive axes, respectively, of the main component 1 defined by antimicrobial activity against yeast, explaining 57.79% of the total variance. The *M. sylvestris* extracts were associated with the main component 2 (13.11% of the total variance), with the positive part defined by antimicrobial activity against *B. cereus* bacteria.

Among the observed results, it can be highlighted that the extracts obtained by ultrasound from the plants belonging to the genera *Cistus*, especially *C. ladanifer* and *C. albidus*, and *Quercus* (*Q. ilex*), were the ones that presented the highest number of phenolic compounds, and highest antioxidant and antimicrobial activity. It was also possible to observe that the extracts of the genus *Cistus*, highlighting *C. ladanifer* extracted by ultrasound, were the ones that presented the best results for antihypertensive activity, since the concentration (µg/mL) of extract necessary to inhibit 50% of ACE is lower, which explains why the IC50 is in the opposite plane for these plant extracts.

## 4. Conclusions

In general, when the two extraction methods used to obtain phenolic compounds were compared, it was found that there were significant differences, with the highest values found for ultrasound extraction. In addition, the extracts obtained from *Cistus albidus*, *C. salviifolius*, *R. ulmifolius* and *Q. ilex* were the ones with the highest concentrations of phenolic compounds. The antioxidant activity was higher in the *Cistus* and *Q. ilex* extracts obtained by ultrasound, as was the antihypertensive activity. The antimicrobial activity was also higher in the extracts obtained by ultrasound from *C. salviifolius* and *Q. ilex* plants against bacteria and *C. ladanifer* against yeast.

Therefore, it can be concluded that, by ultrasound extraction of the phenolic compounds from the plants *C. ladanifer*, *C. albidus* and *Q. ilex*, it is possible to obtain extracts with significant functional properties, so that they could be studied for their use in food with the aim of obtaining healthy and safe products, favouring the sustainability of the Dehesa Extremeña environment.

## Figures and Tables

**Table 1:** Total phenolic compounds of plants expressed in mg of gallic acid equivalents (GAE)/100 g fresh plant.

Plant	Agitation	Ultrasound	*p* ^2^
	Mean SD ^1^	Mean SD	
*Calendula officinalis*	276.73 ± 13.41 ^cde^	95.46 ± 2.24 ^hi^	**<0.001**
*Rosmarinus officinalis*	160.72 ± 14.93 ^de^	314.01 ± 14.65 ^fghi^	**0.001**
*Cistus ladanifer*	802.89 ± 45.52 ^ab^	655.05 ± 62.55 ^cdef^	**0.03**
*Cistus multiflorus*	408.66 ± 52.27 ^cde^	514.26 ± 39.86 ^fgh^	0.08
*Cistus albidus*	580.25 ± 93.34 ^bc^	1260.47 ± 472.40 ^a^	0.152
*Cistus salviifolius*	554.8 ± 59.73 ^bc^	1029.99 ± 140.38 ^ab^	**0.048**
*Lavandula stoechas*	1029.25 ± 23.15 ^a^	554.07 ± 13.66 ^efg^	**<0.001**
*Crataegus monogyna*	387.96 ± 29.43 ^cd^	855.23 ± 18.67 ^bcde^	**<0.001**
*Malva sylvestris*	67.00 ± 9.33 ^de^	156.32 ± 6.69 ^hi^	**0.001**
*Rubus ulmifolius*	305.24 ± 8.79 ^cde^	953.61 ± 76.27 ^abc^	**<0.001**
*Quercus ilex*	387.54 ± 36.58 ^cd^	927.84 ± 112.79 ^abcd^	**0.001**
*Morus alba*	26.95 ± 3.92 ^de^	286.38 ± 10.07 ^ghi^	**<0.001**
*Ulmus* sp.	131.71 ± 22.41 ^de^	263.36 ± 3.17 ^ghi^	**0.001**
*Asparagus* sp.	92.08 ± 4.19 ^de^	302.57 ± 18.61 ^fghi^	**<0.001**
*Urginea maritima*	7.95 ± 5.21 ^e^	57.94 ± 3.71 ^i^	**<0.001**

^1^ SD: standard deviation. ^2^ *p* values of the variable extraction methods. ^a–i^ Values with different superscript letters are significantly different (*p* < 0.05) between plants.

**Table 2:** Antioxidant activity (DPPH and ABTS) expressed in mg Trolox/100 g fresh plant and IC50 values (µg/mL) of ACE inhibitory activity of extracts for the different extraction methods (mechanical agitation (AE) and ultrasound-assisted extraction (UE) performed).

Plant	DPPH		*p* ^1^	ABTS		*p*	IC50		*p*
	AE	UE		AE	UE		AE	UE	
*Calendula officinalis*	60.51 ^c^	183.69 ^c^	**<0.001**	67.05 ^f^	335.94 ^e^	**<0.001**	180.19 ^bcd^	153.85 ^ab^	0.418
*Rosmarinus officinalis*	100.98 ^c^	95.75 ^c^	0.57	256.66 ^e^	273.40 ^ef^	**<0.001**	139.91 ^de^	115.86 ^ab^	0.234
*Cistus ladanifer*	2397.47 ^a^	2363.34 ^a^	**0.015**	467.93 ^d^	753.75 ^bc^	**<0.001**	11.27 ^f^	5.85 ^d^	**0.056**
*Cistus multiflorus*	793.67 ^bc^	953.12 ^bc^	**0.007**	374.72 ^de^	504.78 ^d^	**<0.001**	187.13 ^bcd^	65.46 ^cd^	**0.008**
*Cistus albidus*	276.51 ^c^	262.64 ^c^	0.061	434.68 ^d^	825.51 ^b^	**<0.001**	30.37 ^f^	21.69 ^d^	0.322
*Cistus salviifolius*	40.27 ^c^	100.80 ^c^	**<0.001**	804.74 ^b^	832.72 ^b^	0.305	62.13 ^ef^	16.53 ^cd^	0.107
*Lavandula stoechas*	18.93 ^c^	91.23 ^c^	**<0.001**	45.74 ^f^	256.32 ^f^	**<0.001**	172.47 ^bcd^	189.79 ^a^	0.362
*Crataegus monogyna*	106.25 ^c^	216.46 ^c^	**0.001**	532.99 ^cd^	718.05 ^bc^	**0.012**	174.61 ^bcd^	151.77 ^ab^	0.267
*Malva sylvestris*	37.65 ^c^	147.53 ^c^	**0.001**	77.40 ^f^	262.93 ^f^	**<0.001**	202.73 ^bc^	112.92 ^ab^	**0.039**
*Rubus ulmifolius*	93.28 ^c^	131.50 ^c^	**<0.001**	644.23 ^c^	842.33 ^b^	**0.001**	315.58 ^a^	65.40 ^cd^	**0.036**
*Quercus ilex*	1531.95 ^b^	1611.59 ^b^	0.599	2531.41 ^a^	2306.17 ^a^	0.117	198.58 ^bc^	80.38 ^bc^	**0.001**
*Morus alba*	29.45 ^c^	255.42 ^c^	**<0.001**	60.92 ^f^	303.40 ^ef^	**<0.001**	232.66 ^b^	137.89 ^ab^	**0.042**
*Ulmus* sp.	70.76 ^c^	366.43 ^c^	**<0.001**	261.76 ^e^	397.37 ^e^	**<0.001**	142.77 ^de^	72.63 ^bc^	0.055
*Asparagus* sp.	40.32 ^c^	244.87 ^c^	**0.013**	99.46 ^f^	260.48 ^f^	**0.002**	110.86 ^de^	99.37 ^bc^	0.412
*Urginea maritima*	17.05 ^c^	132.08 ^c^	0.05	41.96 ^f^	154.62 ^f^	**0.002**	198.37 ^bc^	152.02 ^ab^	**0.032**

Values are given as mean (*n* = 3). ^1^ *p* values of the variable extraction methods. ^a–f^ Values with different superscript letters are significantly different (*p* < 0.05) between plants.

**Table 3:** Diameter of inhibition zones in mm of different concentrations of plant extracts tested that showed activity against bacteria.

	*L. Monocytogenes*	*L. Innocua*	*S. Aureus*	*B. Cereus*	*E. Coli*	*S. Choleraesuis*
Plant	Mean SD	Mean SD	Mean SD	Mean SD	Mean SD	Mean SD
*Cistus multiflorus*	-	-	-	-	-	5.26 ± 4.68 ^c^
*Cistus albidus*	1.62 ± 2.40 ^d^	2.89 ± 4.28 ^d^	3.19 ± 4.72 ^d^	2.62 ± 3.87 ^c^	2.35 ± 3.47 ^g^	5.28 ± 2.80 ^c^
*Cistus ladanifer*	1.85 ± 2.76 ^c^	3.23 ± 4.78 ^c^	3.22 ± 3.57 ^d^	3.00 ± 4.46 ^b^	2.86 ± 4.24 ^e^	7.82 ± 2.97 ^a^
*Cistus salviifolius*	0.83 ± 1.93 ^g^	5.29 ± 4.10 ^a^	3.28 ± 3.56 ^d^	-	4.25 ± 3.28 ^b^	4.22 ± 3.79 ^d^
*Lavandula stoechas*	1.38 ± 3.21 ^e^	-	1.49 ± 3.48 ^f^	-	-	0.87 ± 2.03 ^i^
*Quercus ilex*	3.88 ± 4.69 ^a^	3.56 ± 4.04 ^b^	5.45 ± 2.97 ^a^	3.79 ± 3.34 ^a^	5.07 ± 3.86 ^a^	6.27 ± 3.55 ^b^
*Rosmarinus officinalis*	1.15 ± 2.69 ^f^	-	1.88 ± 2.91 ^e^	0.99 ± 2.32 e^f^	0.00 ± 0.00 ^k^	-
*Rubus ulmifolius*	2.18 ± 3.23 ^b^	2.76 ± 3.56 ^d^	3.98 ± 3.08 ^c^	1.89 ± 2.81 ^d^	3.93 ± 2.93 ^c^	3.57 ± 2.06 ^f^
*Crataegus monogyna*	-	-	1.85 ± 2.76 ^e^	-	3.75 ± 3.98 ^d^	-
*Asparagus* sp.	-	-	-	-	2.00 ± 2.95 ^h^	-
*Malva sylvestris*	-	-	4.3 ± 6.60 ^b^	3.88 ± 5.73 ^a^	2.56 ± 3.79 ^f^	3.37 ± 4.98 ^f^
*Morus alba*	-	-	-	-	-	3.85 ± 5.69 ^e^
*Urginea maritima*	-	-	1.18 ± 2.76 ^g^	1.04 ± 2.44 ^e^	1.37 ± 3.19 ^i^	1.52 ± 3.54 ^g^
*Ulmus* sp.	-	-	1.16 ± 2.71 ^g^	0.86 ± 2.00 ^f^	1.18 ± 2.76 ^j^	1.19 ± 2.78 ^g^
**Extraction method**	-					
Agitation	0.63 ± 1.99	0.85 ± 2.53	2.30 ± 3.41	1.38 ± 3.00	2.19 ± 3.33	2.66 ± 3.78
Ultrasound	1.21 ± 2.71	1.68 ± 3.35	2.13 ± 3.77	1.20 ± 2.93	1.99 ± 3.27	3.51 ± 4.18
**Concentration (mg/mL)**						
2	2.10 ± 3.33 ^a^	2.44 ± 4.12 ^a^	4.95 ± 4.44 ^a^	3.39 ± 4.18 ^a^	4.34 ± 3.86 ^a^	5.76 ± 4.80 ^a^
1	0.51 ± 1.85 ^b^	0.95 ± 2.38 ^b^	1.36 ± 2.41 ^b^	0.40 ± 1.48 ^b^	1.75 ± 2.82 ^b^	2.36 ± 3.07 ^b^
0.5	0.15 ± 0.80 ^c^	0.41 ± 1.49 ^c^	0.34 ± 1.24 ^c^	0.08 ± 0.42 ^c^	0.19 ± 0.99 ^c^	1.14 ± 2.09 ^c^
*Pplants*	<0.001	<0.001	<0.001	<0.001	<0.001	<0.001
*Pextraction*	<0.001	<0.001	<0.001	<0.001	<0.001	<0.001
*Pconcentration*	<0.001	<0.001	<0.001	<0.001	<0.001	<0.001
*Pplant × extraction*	<0.001	<0.001	<0.001	<0.001	<0.001	<0.001

-: no inhibition; ^a–k^: values with different superscript letters indicate statistical differences (*p* < 0.05).

**Table 4:** Diameter of inhibition zones in mm of different concentrations of plant extracts tested that showed activity against yeasts.

Plant	*C. Boidinii*	*K. Fluxuum*	*P. Carsonii*	*Z. Bailii*
	Mean SD	Mean SD	Mean SD	Mean SD
*Cistus albidus*	2.83 ± 4.2 ^d^	5.17 ± 4.11 ^b^	4.87 ± 3.76 ^c^	5.31 ± 3.04 ^c^
*Cistus ladanifer*	7.55 ± 2.7 ^a^	7.83 ± 3.21 ^a^	8.11 ± 3.08 ^a^	7.69 ± 2.93 ^a^
*Cistus salviifolius*	1.62 ± 3.79 ^e^	4.53 ± 4.04 ^bc^	-	1.31 ± 3.06 ^d^
*Lavandula stoechas*	-	1.54 ± 2.98 ^d^	2.33 ± 3.50 ^d^	1.33 ± 3.09 ^d^
*Quercus ilex*	5.46 ± 6.09 ^b^	7.53 ± 4.89 ^a^	3.37 ± 4.65 ^b^	6.52 ± 5.03 ^b^
*Rosmarinus officinalis*	-	1.025 ± 2.39 ^d^	1.2 ± 2.80 ^e^	-
*Rubus ulmifolius*	3.15 ± 4.89 ^c^	3.51 ± 3.71 ^c^	1.01 ± 2.37 ^f^	1.16 ± 2.71 ^e^
**Extraction method**				
Agitation	1.87 ± 3.57	3.62 ± 3.41	2.76 ± 3.73	3.24 ± 4.14
Ultrasound	4.02 ± 5.04	5.27 ± 5.01	3.78 ± 4.43	3.42 ± 4.26
**Concentration (mg/mL)**				
2	5.97 ± 5.53 ^a^	7.05 ± 5.26 ^a^	5.88 ± 4.87 ^a^	6.12 ± 4.89 ^a^
1	2.14 ± 3.53 ^b^	4.79 ± 2.83 ^b^	3.20 ± 3.31 ^b^	2.87 ± 3.46 ^b^
0.5	0.73 ± 1.83 ^c^	1.51 ± 2.49 ^c^	0.73 ± 1.82 ^c^	0.99 ± 1.96 ^c^
*Pplants*	<0.001	<0.001	<0.001	<0.001
*Pextraction*	<0.001	<0.001	<0.001	<0.001
*Pconcentration*	<0.001	<0.001	<0.001	<0.001
*Pplant × extraction*	<0.001	<0.001	<0.001	<0.001

-: no inhibition; ^a–f^: values with different superscript letters indicate statistical differences (*p* < 0.05).

## Data Availability

Not applicable.
